# Genomic Location of the Major Ribosomal Protein Gene Locus Determines *Vibrio cholerae* Global Growth and Infectivity

**DOI:** 10.1371/journal.pgen.1005156

**Published:** 2015-04-13

**Authors:** Alfonso Soler-Bistué, Juan A. Mondotte, Michael Jason Bland, Marie-Eve Val, María-Carla Saleh, Didier Mazel

**Affiliations:** 1 Institut Pasteur, Unité Plasticité du Génome Bactérien, Département Génomes et Génétique, Paris, France; 2 Centre National de la Recherche Scientifique UMR3525, Paris, France; 3 Institut Pasteur, Viruses and RNA Interference; 4 Centre National de la Recherche Scientifique UMR3569, Paris, France; Universidad de Sevilla, SPAIN

## Abstract

The effects on cell physiology of gene order within the bacterial chromosome are poorly understood. *In silico* approaches have shown that genes involved in transcription and translation processes, in particular ribosomal protein (RP) genes, localize near the replication origin (*oriC*) in fast-growing bacteria suggesting that such a positional bias is an evolutionarily conserved growth-optimization strategy. Such genomic localization could either provide a higher dosage of these genes during fast growth or facilitate the assembly of ribosomes and transcription foci by keeping physically close the many components of these macromolecular machines. To explore this, we used novel recombineering tools to create a set of *Vibrio cholerae* strains in which *S10-spec-α* (S10), a locus bearing half of the ribosomal protein genes, was systematically relocated to alternative genomic positions. We show that the relative distance of S10 to the origin of replication tightly correlated with a reduction of S10 dosage, mRNA abundance and growth rate within these otherwise isogenic strains. Furthermore, this was accompanied by a significant reduction in the host-invasion capacity in *Drosophila melanogaster*. Both phenotypes were rescued in strains bearing two S10 copies highly distal to *oriC*, demonstrating that replication-dependent gene dosage reduction is the main mechanism behind these alterations. Hence, S10 positioning connects genome structure to cell physiology in *Vibrio cholerae*. Our results show experimentally for the first time that genomic positioning of genes involved in the flux of genetic information conditions global growth control and hence bacterial physiology and potentially its evolution.

## Author Summary

Increasing evidence indicates that nucleoid spatiotemporal organization is crucial for bacterial physiology since these microorganism lack compartmentalized nucleus. However, it is still unclear how gene order within the chromosome can influence cell physiology. Here, by systematically relocating ribosomal protein genes to different genomic positions in *Vibrio cholerae*, we revealed drastic differences in growth rate and infectivity of this isogenic strain set. We show that genomic positioning of ribosomal protein genes is crucial for physiology by providing replication-dependent higher dosage. Therefore it might play a key role in genome evolution of bacterial species. This work will contribute to discover genomic rules governing cell physiology which will be essential in the context of the creation of new artificial life forms.

## Introduction

The bacterial genome consists of a DNA molecule which is compacted 1000-fold to occupy about 15% of the cell volume [1]. The genome is precisely organized within such a limited cellular space to ensure that DNA replication, segregation and gene expression are well orchestrated [1–3]. Bioinformatics studies suggest that gene order within the chromosome contribute to the spatial organization of the DNA molecule and may optimize bacterial physiology [4, 5]. Despite the insight brought by such *in silico* studies, experimental evidence is scarce [6–11].

Bacterial genomes are very flexible with respect to their gene repertoire but display highly conserved organizational features at the sequence level that deeply impact cell physiology [12, 13]. An important organizational characteristic is the existence of a single origin of replication (*oriC*), where DNA duplication begins, proceeding unidirectionally through two equally sized replichores up to the chromosomal terminal region [12]. This establishes interplay between genome structure and cell physiology. For example, essential genes tend to be in the replicative leading strand to avoid head-on collisions between the replication and transcription machineries [14]. Also, in optimal growth conditions, some bacteria divide faster than the estimated time required for whole genome duplication. To solve this, fast-growing microorganisms fire their *oriC* multiple times before cell division leading to simultaneous replication rounds [12, 15]. As a consequence, genes close to *oriC* transiently benefit from higher dosage.

Comparative genomic studies revealed that in fast-growing bacteria, essential genes involved in the expression of genetic information such as RNA polymerase (RNAP), ribosomal genes and specific tRNAs are found near *oriC* [16]. In parallel, it has been observed that both ribosome and RNA polymerase molecule counts can vary by one order of magnitude according to the cell physiological state, reaching its peak at the exponential phase. This is when the growth rate is maximal and bacteria need the highest synthesis capacity [17]. In these circumstances, ribosomes account for 30% of the cell’s dry mass [18]. Therefore, positional bias of ribosomal and RNAP genes could provide the advantage of a higher dosage during fast-growth [12]. Such a hypothesis, although plausible for rRNA operons, could not be appropriate for RP and RNAP transcripts as they are not the final functional products. As protein coding genes, they possess several regulation mechanisms [19–24] that can easily buffer any putative gene dosage difference given by replication-dependent gene dosage effects. Additionally the fact that rRNA are in multiple copies enhances replication-linked gene dosage effect. For example, *Escherichia coli* bears 7 rRNA copies, therefore in exponential phase their copy number can go from 7 to 28 while RP and RNAP which are in single copy can go from 1 to as most 4 copies. On the other hand, gene expression can vary by 300-fold according to its genomic location, an amount that is not explained by gene dosage effects but rather by the presence of overlapping chromosome structural organization features [8, 25]. Alternatively to the gene-dosage hypothesis, the bias in the genomic location of this genes might reflect a constraint for the optimal and fast assembly of the ribosomes subunits and RNAP which would necessitate these genes in close proximity within the crowded cellular space [3, 9, 26, 27].

The experimental link between RNAP and ribosomal gene location, and bacterial growth rate is missing. *Vibrio cholerae*, the etiological agent of cholera is a unique model to test this link. It is among the fastest-growing bacteria, displaying one of the highest number of simultaneous replication rounds [16]. In addition, it is a model for the study of bacteria with multiple chromosomes. Its genome consists of a main chromosome (Chr1) of 2.96 Mbp which harbors the majority of essential genes and a second chromosome (Chr2) of 1.07 Mbp that encodes a higher proportion of hypothetical genes [28]. Genome replication starts at *oriC* of Chr1 (*oriC1*) while *oriC* of Chr2 (*oriC2*) fires only when the two thirds of the larger replicon have already been duplicated. As a consequence both chromosomes finish their replication synchronously [29]. Genes on Chr1 are on average more expressed than those in Chr2 [7, 30]. The ribosomes are encoded by 8 rRNA operons and more than 50 different RP genes that are in single copy. Previous studies dealing with ribosomal genes focused on rRNAs, whose copy number, regulation and mechanism of action drastically differs from RP. Indeed experimental evidence established connections between rRNA copy number and orientation, to cell physiology [31–36]. The role of RP genomic positioning has been overlooked since it is difficult to work with essential genes that are spread along the genome and in single copy.

The *s10-spec-α* (there after called S10) is a 13.2 Kbp locus harboring half of RP genes, *secY* and the gene coding the RNAP α-subunit, that is highly conserved in archaea, bacteria and eukaryotes[24]. It is always located in bacterial primary chromosomes [37, 38]. In *V*. *cholerae*, all their genes are essential [39].Taking advantage of S10 proprieties, we decided to use it as a model to assess the link between the genomic location of RP genes and cell physiology. We applied a “positional genetics” approach by gradually moving S10 away from its original location using recombineering techniques without widely affecting genome order. We measured the growth rate (GR), the S10 dosage and expression levels of these derivatives in fast-growing conditions. We showed that GR diminished in a distance-related manner while S10 dosage and mRNA abundance closely correlated this trend. Some of these derivatives displayed an impaired infection capacity *in vivo*, using *Drosophila melanogaster*. Importantly, the strains bearing two copies of S10 far away from *oriC1* displayed restored GR and host-invasion capacity demonstrating that gene dosage is the main mechanism behind the behavior of the mutant strains. Our study provides strong evidence supporting the interplay between gene order and cell physiology. Genomic positioning can be seen as a regulation mechanism for RP, having a critical role in bacterial physiology as observed for global growth control. Indeed, S10 repositioning allows us to fine tune GR in *V*. *cholerae* with a mere relocation of a 13.2 Kbp locus. The advantages conferred during host invasion and its positional flexibility contrasted to its conserved location among *Vibronaceae* suggesting that S10 location might play a role in the evolutionary success of *V*. *cholerae*.

## Results

### Analysis of S10 genomic location among *Vibrionaceae*

In *V*. *cholerae*, as in other fast-growing organisms, ribosomal genes are positioned around the *oriC1* (S1 Fig). Among 56 RP genes in the genome, 26 are included within S10 (S1 and S2 Tables). All S10 genes are encoded in the replication leading strand. The analysis of 18 genomes representing the 3 genera and 12 species representatives of the *Vibronaceae* family shows that S10 genomic location is conserved close to the *oriC1* among the whole clade (S3 Table). Hence, the S10 locus of *V*. *cholerae* is an adequate model to inquire possible interplay between genomic location of RP genes and cell physiology.

### Recombineering permits targeted relocation of the *s10-spec-α* locus

**Fig 1:**
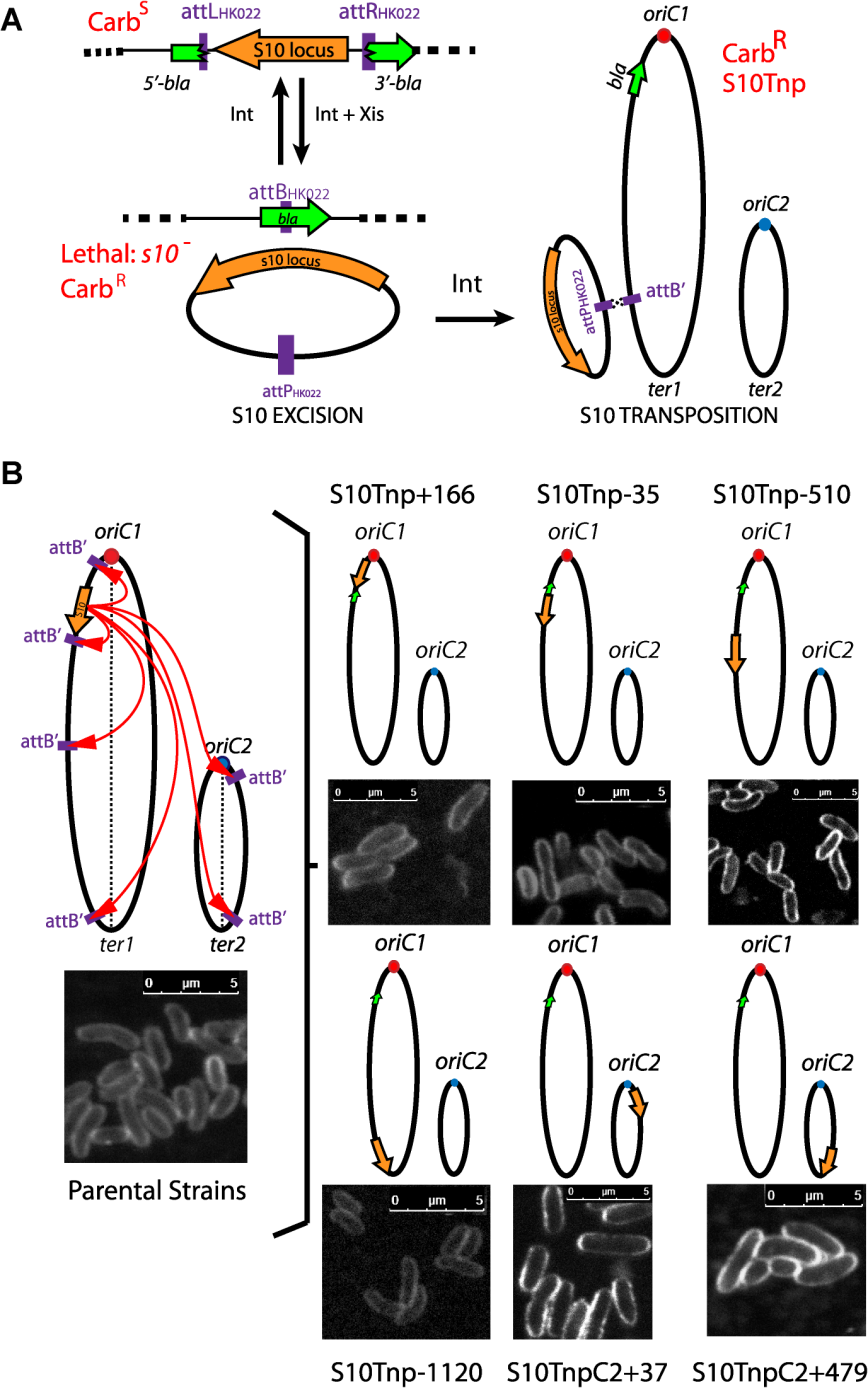
Generation of S10Tnp strains. **(a)** S10 is moved by flanking it by HK022 attL and attR sites. Upon transient expression of phage recombinases a DNA circle containing S10 is excised and *bla* reporter is reconstituted. Viable carbenicillin resistant (Carb^R^) cells are obtained if S10 reintegrates at attB’. **(b)** The obtained S10Tnp strains: ellipses represent chromosomes while small dots represent origin of replication of Chr1 (*oriC1*,red) and Chr2 (*oriC2*, blue). Orange arrows depict S10 position within the genome. The green arrow shows *bla*. Left panel, picture representative of one of the parental strains from which S10Tnp derivatives were produced using attB’ sites at different positions. Right panel, the derivatives showing S10 relocation are shown. Insets correspond to CLSM images of each strain stained with FM5-95, the white bar represents 5 μm (see Supporting Information).

To test S10 repositioning effects on bacterial physiology, we decided to relocate this 13.2 Kbp locus at different positions along Chr1: closer to *oriC1*, next to its original position, to the middle of the replichore and to the terminal region. Secondly, we also sought to move S10 to Chr2. We employed recombineering tools that have been developed for precise genomic remodeling [40, 41]. Using natural transformation [42], the S10 locus was surrounded by attL and attR sites from phage HK022 flanked respectively by the 5’ and 3’ parts of the β-lactamase gene (*bla*) (Fig 1A). Then, an attB site (attB’) was inserted at the chosen site for S10 relocation (Fig 1B and S4 Table). Upon transient expression of the Int and Xis recombinases, attL X attR recombination led to excision of a DNA circle [40] carrying S10 and the attP site while reconstituting the *bla* reporter gene (Fig 1A, Exicision). Since cells lacking S10 are not viable, reintegration events by attP X attB’ recombination, were selected on carbenicillin supplemented medium (Fig 1A, Transposition). Orientation of attB’ ensured S10 co-linearity with the replication fork, avoiding replication-transcription conflicts [14]. We used *bla* instead of *lacZ* as a reporter because it allowed for an easy selection of low frequency transposition events with minimal subculturing, avoiding parental strain contamination and the emergence of suppressor clones. The use of *bla* required shortening the core-binding sequence [43] and insertion of a point mutation in the embedded attB site, this was done to prevent frame shifts mutations and premature stop codons (see S1 Text). As a consequence, S10 transposition was favored over reinsertion (Fig 1A), since the attB’ sequence is wild type while the attB sequence within *bla* is altered.

Using this strategy, S10 was moved from its original location along the left replichore 166 Kbp towards *oriC1* and 35, 510, 1120 Kbp towards the end of Chr1. In parallel, it was also relocated to Chr2, near *oriC2* and close to the terminus (Fig 1B and S4 Table). We refer to these mutants using the locus name (S10), followed by Tnp (for transposition), and the distance in Kbp from its original location. Minus and plus refer to up or downstream movements within the genome sequence. For relocations to Chr2, C2 was added while the numbers correspond to the distance from *oriC2* (Fig 1B). Notably, the derivatives display no viability loss and show normal cell morphology (Figs 1B and S2). The resulting isogenic strain set allows for studying the possible effects of S10 relocation. Overall, S10 locus relocation was well tolerated. Since S10 position is conserved along *Vibronaceae* family, this also suggests that its current location is the result of selective pressure along the evolution of this clade (S3 Table).

### S10 relocation generates a distance-dependent growth rate reduction

**Fig 2:**
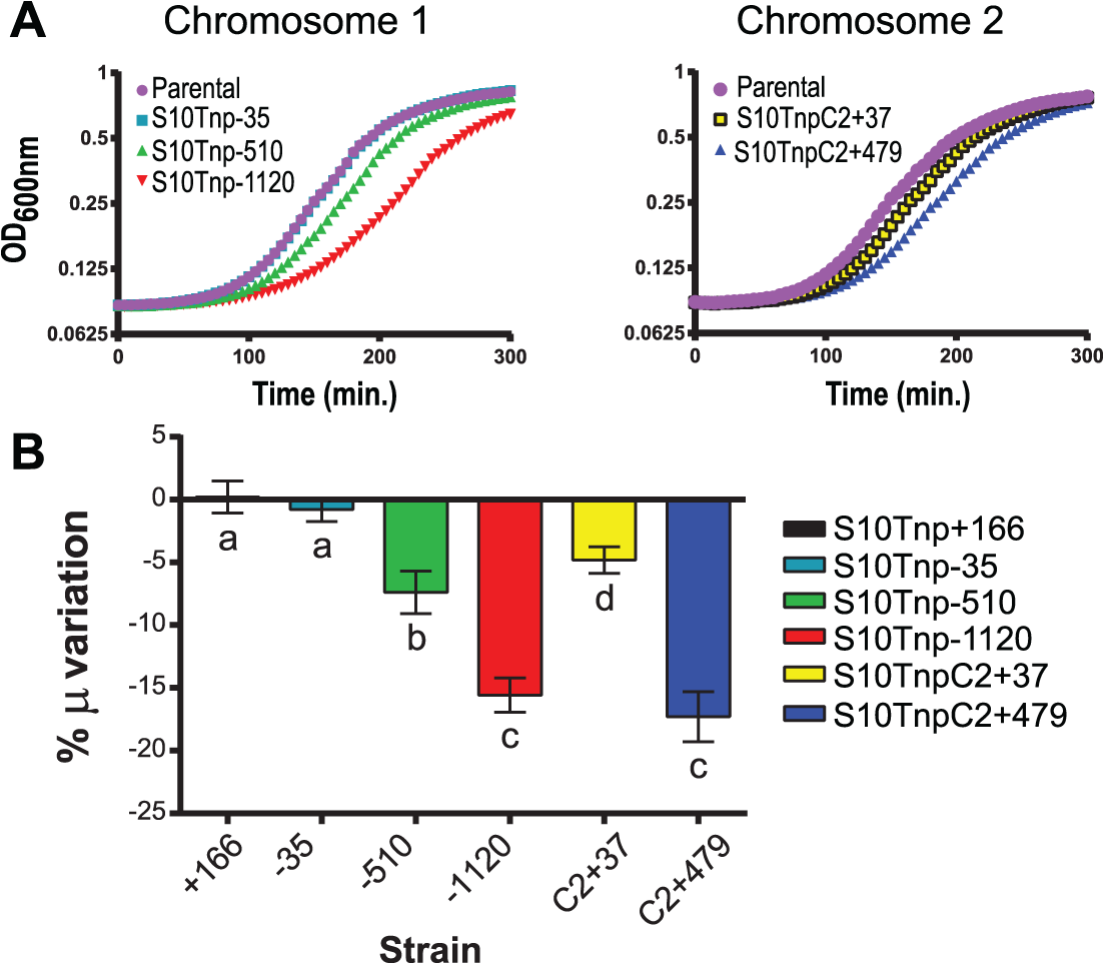
Growth rate diminishes in S10Tnp mutants in a distance-related manner. **(a)** Representative growth curve in fast-growth conditions performed as described in Supporting Information. **(b)** S10 relocation effect on GR was quantified by averaging obtained μ in at least 5 independent experiments for each S10Tnp strain and normalizing it to the μ of the parental strain. Results are expressed as percentage of the variation (μ %) with 95% CI with respect to parental strains. Statistical significance was analyzed by one-way ANOVA two-tailed test. Then Holm-Sidak test was done to compare the means values obtained for each strain. Letters denote groups being statistically different.

Former bioinformatics studies have correlated short generation times to proximity of RP and RNAP genes to *oriC*, suggesting that this could provide a higher dosage of these genes during fast growth [16]. If this is the case, in fast-growing conditions, delayed growth should be observed, the further away S10 is relocated from *oriC*. To test this, we measured GR of all strains in fast-growing conditions, using the slope of the growth curve during exponential phase (μ). All parental strains displayed similar GR (S3 Fig), indicating that the attB’ site insertion did not interfere with cell physiology. The S10Tnp-35 strain, where S10 location is slightly changed, displayed no μ alteration compared to the parental strain, showing that S10 precise position is not important for optimal growth and that the transposition process by itself exerts no influence on GR (Fig 2 and S5 Table). The GR of strain S10Tnp+166, where S10 was placed in close proximity to *oriC1*, did not significantly differ from the parental strain. Meanwhile, strains S10Tnp-510 and S10Tnp-1120 showed μ reductions of 7.39 ± 2.67% and 15.58 ± 3.14% respectively (Fig 2B). Therefore, increasing distance between S10 and *oriC* of Chr1 (*oriC1*) correlated with greater μ differences. S10 transpositions to Chr2 caused significant μ reductions of 4.8 ± 1.8% and 17.29 ± 3.44% for S10TnpC2+37 and S10TnpC2+479 respectively. Hence, correlation between distance of relocation and GR differences is also observed for this replicon (Fig 2 and S5 Table).

GR measurements of S10Tnp mutants in slow-growing conditions showed no significant μ reduction with respect parental strains (S8 Table and S4 Fig). We infer that in slow-growing conditions genome replication can be completed before cell division hence, simultaneous replication rounds do not occur, greatly reducing gene dosage differences [7, 44].

Since optical density curves used for growth measurements rely on indirect estimation of the population by turbidimetry, they cannot distinguish if the observed μ differences are due to slower growth, aberrant division or the death of a subpopulation of daughter cells. Therefore, we followed bacterial division of each strain using time-lapse microscopy in fast-growing conditions. Images of parental and S10Tnp derivative strains taken every 2 minutes (S1–S7 Videos) showed that bacterial division proceeds normally in all cases. Therefore, GR differences are a consequence of a slower population growth.

In summary, we obtained a set of isogenic strains displaying different GR phenotypes. In fast-growing conditions, a distance-dependent GR reduction was observed upon S10 repositioning. Interestingly, there were distance dependent differences along Chr2 suggesting gene-dosage effects within this replicon. In slow-growth conditions the strains showed no GR variation.

### S10 genomic position determines its dosage and expression

**Fig 3:**
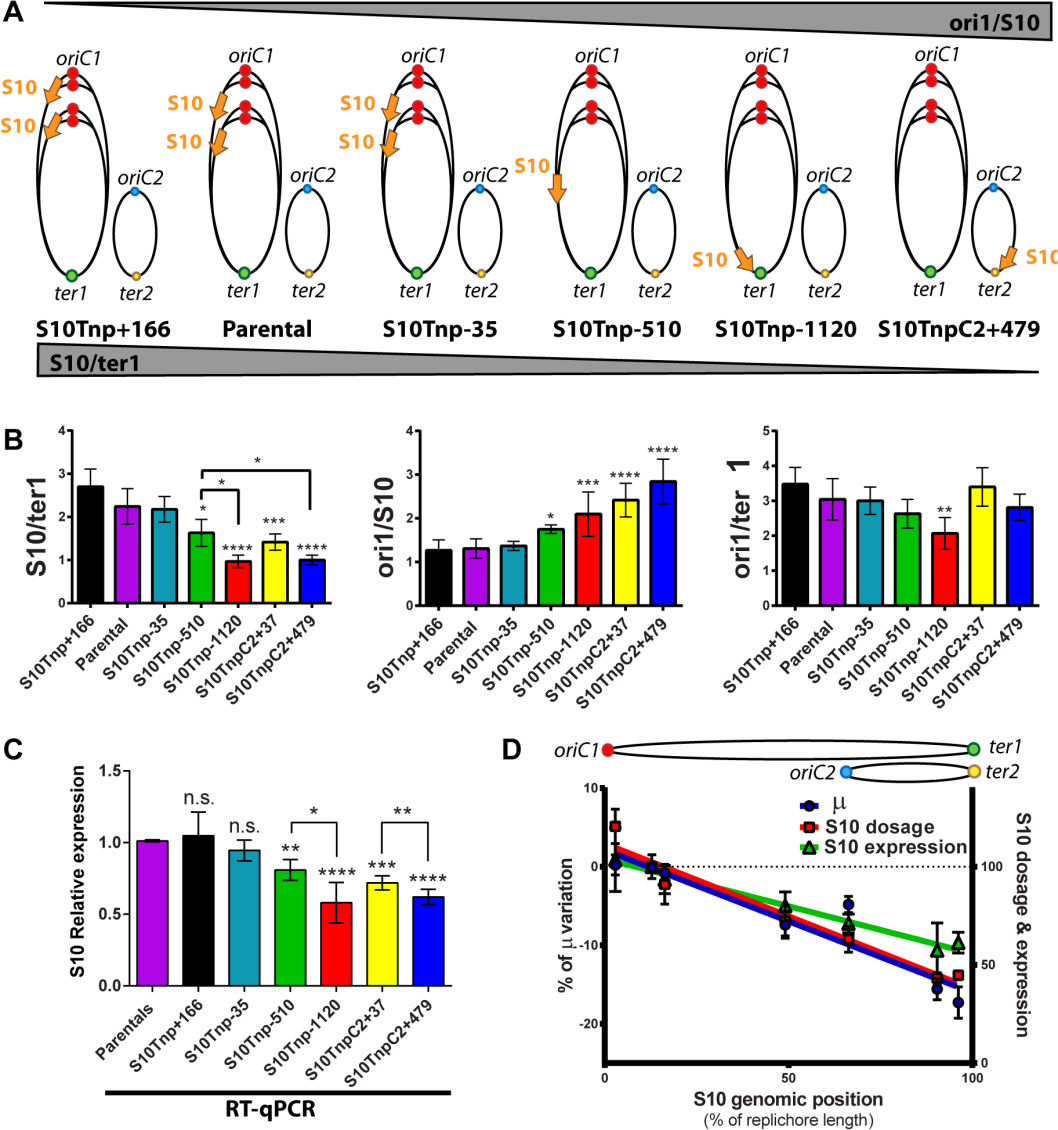
S10 dosage and expression diminish in a distance-related manner correlating with GR reduction. **(a)** Expected trend on S10/ter1 and ori1/S10 ratios according to locus repositioning. Ellipses represent chromosomes. Colored dots depict *oriC1* and *oriC2* and termini of Chr1 (*ter1*) and Chr2 (*ter2*). Simultaneous replication rounds are shown. An orange arrow represents the S10 locus. The expected trend for ori1/s10 and s10/ter1 is shown by top and bottom triangles. **(b)** Gene dosage measurements obtained by qPCR in fast-growth conditions. **(c)** S10 expression normalized to parental strains obtained by RT-qPCR. **b** and **c** show the mean and error bars representing 95% CI. Statistical significance was assessed by one-way ANOVA two tailed test and Tukey test for multiple comparisons. n.s. stands for non-significant, p>0.05; *, p<0.05; **, p<0.01; ***, p<0.001;****, p<0.0001. **(d)** S10 dosage (red), expression (green) and % variation of μ (blue) of each bacterial strain were plotted as a function S10 position within the genome measured as % of replichore length. Linear regression is plotted for each variable. Chr2 was overlapped to Chr1 according to cell cycle order. Chromosomes are schematized on the top of the graph.

During fast growth, genes positioned near the *oriC* transiently exhibit higher dosage [7, 8, 30]. Therefore, S10 relocation far away from its original position should produce a lower dosage. A concomitant reduction of expression would support the gene-dosage hypothesis. To test this, we used quantitative PCR (qPCR) to quantify S10 abundance in fast-growth conditions in our strain set. Replication origins and replication termini (ori1, ori2, ter1 and ter2 respectively) were also quantified to normalize the S10 measurements (Fig 3A). A lower s10/ter1 ratio is to be expected as S10 is moved further away from *oriC1* while the ori1/s10 quotient should increase (Fig 3A).

The s10/ter1 estimates S10 copy-number of S10 since there is one ter1 per cell. For S10 relocations within Chr1, this ratio was the highest for S10Tnp+166, the parental strain and S10Tnp-35 which showed more than two S10 copies per cell (Table 1). Then, it decreases to 1.62 ± 0.31 for S10Tnp-510 and to one copy when S10 is located at the terminus of Chr1 as in S10Tnp-1120 (Table 1 and Fig 3B, left panel). Therefore, S10 dosage is reduced with further relocations within Chr1. For Chr2, S10TnpC2+37 S10/ter1 showed a value of 1.41± 0.18, which is not different from S10Tnp-510 but was significantly higher than the one displayed by S10TnpC2+479 (1.02 ± 0.11). This is consistent with the fact that *oriC2* fires after 2/3 of Chr1’s replication has been completed and both replicons finish synchronously [29]. In S10Tnp-1120 and S10TnpC2+479 there is 1 S10 copy, showing that its dose is twice as low when located at the terminal region of Chr1 or Chr2.

**Table 1:** Gene dosage measurements performed by qPCR experiments on the full strain set.

**Strain**	**S10/ter1**	**ori1/S10**	**ori1/ter1**
S10Tnp+166	2.7 ± 0.40	1.26 ± 0.24	3.47 ± 0.48
Parental	2.24 ± 0.41	1.31 ± 0.22	3.04 ± 0.59
S10Tnp-35	2.17 ± 0.29	1.37 ± 0.10	3.01 ± 0.39
S10Tnp-510	1.6 ± 0.31	1.75 ± 0.09	2.63 ± 0.41
S10Tnp-1120	0.96 ± 0.14	2.09 ± 0.51	2.06 ± 0.45
S10TnpC2+37	1.41 ± 0.19	2.41 ± 0.38	3.39 ± 0.55
S10TnpC2+479	1.00 ± 0.11	2.84 ± 0.51	2.81 ± 0.37

The ori1/s10 ratio relativizes the *oriC1* abundance to the number of S10 copies present in the cell. It was used to estimate S10 dosage while we expected to display the opposite trend than S10/ter1 (Fig 3A). S10Tnp+166, parental and S10Tnp-35 strains displayed similar ratio close to 1.3 (Table 1 and Fig 3B middle panel). The quotient then increased when S10 is moved away from *oriC1* showing a values of 1.75 and 2.09 for S10Tnp-510 and S10Tnp-1120 respectively. As expected, S10TnpC2+37 and S10TnpC2+479 also showed a significantly higher ori1/S10 ratio than the parental strain since S10 was moved to Chr2 (Table 1).In parallel, with lower GRs, a reduction in oriC1/ter1 ratio was expected. S10Tnp-1120 displayed a significant ori1/ter1 reduction (Table 1 and Fig 3B right panel) with respect to the parental strain. Meanwhile, S10Tnp-510 showed a mild reduction, which was not statistically different from the parental strain or S10Tnp-1120. A Post-test for linear trend of ori1/ter1 among strains in which S10 was relocated along Chr1 shows a statistically significant result (p<0.001, slope -0.21, R^2^ = 0.61). Hence, there is a linear trend of ori1/ter1 reduction when S10 is relocated along this chromosome (Fig 3B, right panel and Table 1). Similarly, the ori1/ter1 quotient mean is higher for S10TnpC2+37 than for S10TnpC2+479. However there are not enough values to perform this statistical test for Chr2.

To detect if S10 dose reduction led to changes in its expression we performed reverse transcription coupled to qPCR (RT-qPCR) on RNA extracted from the same samples. Relative S10 mRNA abundance in each mutant was normalized to parental strain values (Fig 3C). S10Tnp+166 and S10Tnp-35 showed no significant expression change (1.05 ± 0.19 and 0.94 ± 0.08). S10Tnp-510 and S10TnpC2+37 showed a significant reduction (0.81 ± 0.05 and 0.72 ± 0.03 respectively). S10 expression reached a minimum for S10Tnp-1120 and S10TnpC2+479 which displayed a 42% (0.58 ± 0.15) and 38% (0.62 ± 0.05) decrease respectively. Expression reductions up-to 50–60% were to be expected if they were a consequence of a lower S10 dosage due to change in its position. The observed reductions were within this limit. On the other hand, changes in expression due to replication-associated gene dosage effect have not been detected on secondary chromosomes therefore we expected no differences for S10 relocations at different positions in Chr2 [7, 30]. However, we detected a gene-dosage effect on Chr2 since S10 expression in S10TnpC2+37 and S10Tnp+479 was significantly different. Such a variation is not explained by an alteration in Chr2’s cycle, since ori2/ter2 remains unchanged and S10/ter2 follows the same trend as the S10/ter1 ratio (S5 Fig).

To detect correlations between the measured parameters, % μ variation, S10 dosage and expression were plotted as a function of S10 position along the replichore (Fig 3D). Upon this analysis a clear linear dependence of the three variables on S10 position emerged (p<0.001, R^2^≥0.9, S6 Table). Then, GR, S10 dosage and mRNA abundance co-variation was computed with a Pearson correlation coefficient (r). Importantly, GR showed a highly significant correlation with S10 dosage (r = 0.91, p<0.01) and mRNA abundance (r = 0,926, p<0.005). S10 expression also displayed a high correlation with dosage (r = 0.984, p<10^–4^).

In sum, our experiments strongly support that S10 genomic positioning determines its dosage and concomitantly influences S10 expression. In turn, these factors determine maximum GR. Additionally, S10 repositioning along the chromosomes caused a gradual decrease in ori1/ter1 ratio suggesting a lower *oriC1* firing frequency.

### S10 dosage reduction is the mechanism behind positioning effects

To show that S10 positioning is sufficient to explain the observed GR reductions, we returned the locus to its original location using a two-step strategy (S6 Fig). We named these derivatives ΔS10Tnp. Then, for each lineage, GRs of S10Tnp and ΔS10Tnp derivatives were compared to the parental strain in fast-growing conditions (S6 Fig and S7 Table). In all cases, GR rate was either partially complemented, as in -510 and C2+37 series or fully restored as in -1120 and C2+479 series. These results demonstrate that the observed effects in GR are due to changes in S10 positioning within *V*. *cholerae* genome. In the case of S10Tnp-510 and S10TnpC2+37, mutations possibly accumulated along the successive genetic modifications performed might have contributed to a slower growth rate.

**Fig 4:**
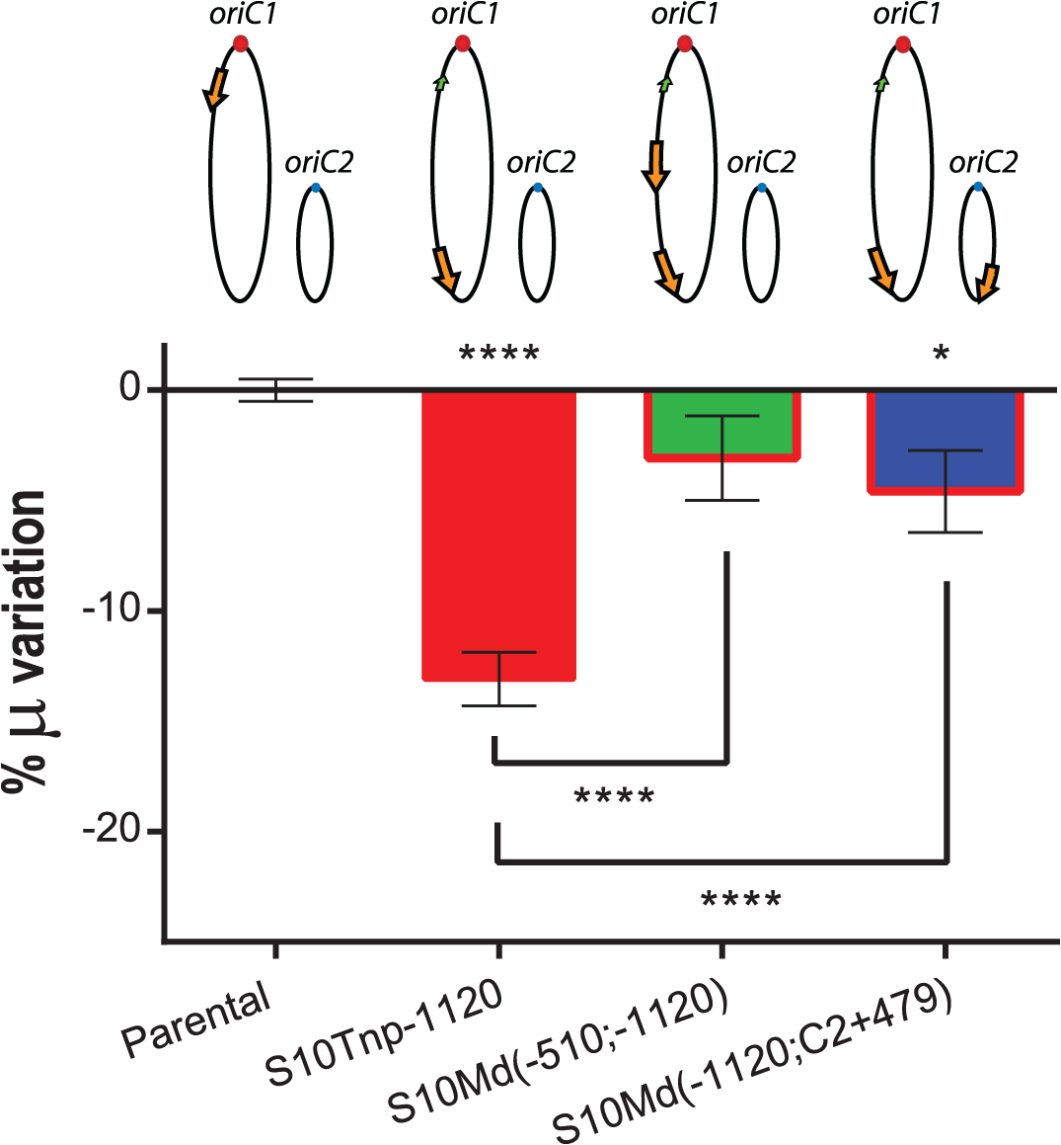
GR defect is consequence of gene dosage reduction. S10 dosage effect was quantified by averaging obtained μ for each strain and normalizing it to the value of the parental strain. Results are expressed as percentage of the variation (μ %) with 95% CI showing complementation of S10Tnp-1120 mutant. Values were obtained from 5 experiments using several independently obtained clones. Statistical significance was assessed by one-way ANOVA two-tailed test. Tukey test was performed for multiple comparisons. n.s. stands for non-significant, p>0.05; *, p<0.05; **, p<0.01; ***, p<0.001;****, p<0.0001.

Physiological effects showed above are most-likely the result of the loss of replication-associated S10 dosage and the consequent reduction in its expression when S10 is relocated far from *oriC1*. However, other mechanisms could also explain the observed GR defects. First, the insertion of such a highly expressed locus could alter chromosome organization which in turn could be detrimental for cell physiology *per se*, independently of S10 mRNA abundance. Second, in the S10Tnp derivatives were the locus was repositioned far away from their original location, S10 position and dosage were altered simultaneously. If S10 products are required in *cis*, the locus repositioning could be detrimental for cell growth simply by physically separating S10 from functional partners such as rRNA operons and other RNAP and RP genes which are located close to *oriC1* (S1 Fig). To test these alternative hypotheses we built merodiploid strains harboring one copy of the locus at the terminal region of Chr1 and a second copy either at middle of the left replichore of Chr1 or at the terminal region of Chr2. For this, S10Tnp-1120 was transformed with genomic DNA from S10Tnp-510 or S10TnpC2+479 (see Supporting Information). These derivatives were called S10Md(-510;-1120) and S10Md(-1120;C2+479) respectively. Since, each S10 copy occupied the same location as in previous experiments, deleterious effects intrinsic to S10 heterologous position should persist. Simultaneously, these two strains have an increased S10 dosage, although both loci copies are far away from their original genomic position. We then compared GR of the parental, S10Tnp-1120, S10Md(-510;-1120) and S10Md(-1120;C2+479) strains in rich medium (Fig 4). S10Tnp-1120 showed a μ reduction of 13.09 ± 2.28%. This effect was abolished by the addition of an extra copy of S10 at the middle of the left replichore of Chr1, since S10Md(-510;-1120) displayed GR similar parental strain (-3.07 ± 3.59%). In parallel, S10Md(-1120;C2+479) showed a slight but significant GR reduction (-4.58 ± 3.48%) when compared to the parental strain (Fig 4). This suggests an incomplete gene dosage complementation when an extra S10 copy is inserted at the terminal region of Chr2. This is supported by the fact that the combined dosages of S10Tnp-1120 and S10Tnp-C2+479 equals 87.93 ± 4.4% of the parental S10 dose (Fig 3B). Complementarily, we computed an S10 dosage of 91.43% of the parental strain when S10Md(-1120;C2+479) % μ variation experimentally observed was introduced into the linear regression equations (S6 Table).

In sum, these experiments demonstrate that S10 position directly influences GR. Since S10 dosage complements this phenotype independently of its location, gene dosage must be the main mechanism behind the observed physiological effect.

### S10 position deeply influences host colonization

The observation that the S10 locus can be relocated within the *V*.*cholerae* genome, made us wonder whether this phenotypic change could be noticeable in a system closer to environmental circumstances, such as a natural infection. We used the fruit fly *Drosophila melanogaster* [45, 46] to quantify putative effect of S10 relocation *in vivo* by measuring *V*.*cholerae* proliferation within flies. This infection model raised great interest because it reproduces cholera symptoms [46], an important feature since mammalian models are often limited to neonatal, chemically or surgically intervened animals. Additionally, insects constitute potential dispersion vectors for the bacterium [47–49].

In our experimental setup, bacterial cultures were diluted in 10% sucrose PBS, a medium not suitable for *V*.*cholerae* growth but allowing fly subsistence. Thus, any bacterial load increase is done at host expense. Feeding insects for 1 hour with this bacterial suspension yielded 10^1^ CFU/fly at the beginning of the experiment. Next, flies were transferred into vials containing plugs embedded in sterile sucrose PBS solution and bacterial burden was measured every 24 h post infection (pi) (S7 Fig). As in previous studies [45], we found a colonization bottle-neck within the first 24 h. Bacterial charge then reached its maximum value at 48 h pi (≈10^4^CFU/fly). The 3 to 4 orders of magnitude increase in bacterial load shows that *V*. *cholerae* is able to colonize and proliferate within the insect. Bacterial load slowly decreases from 48 h on (S7 Fig) and remains detectable at low levels by 10 days pi.

**Fig 5:**
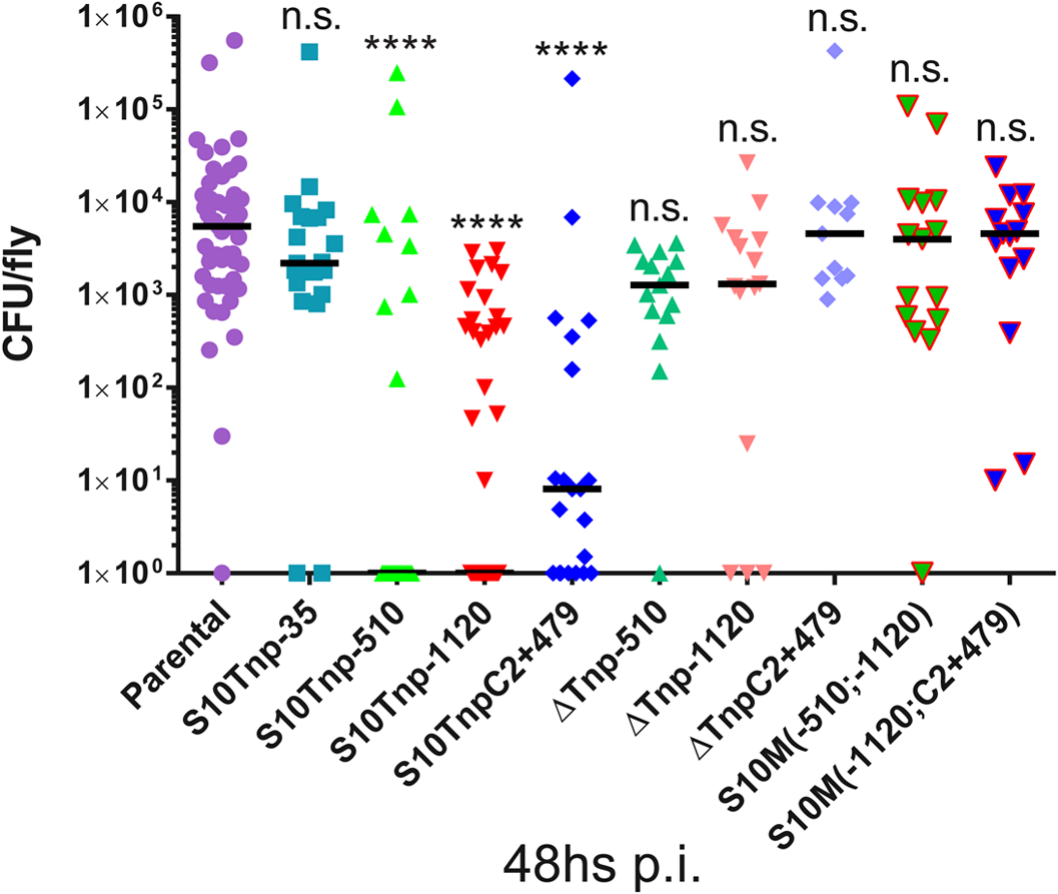
S10-positioning influences *D*. *melanogaster* infection-capacity of *V*. *cholerae*. Bacterial load within flies 48h pi with parental (n = 53), S10Tnp-35 (n = 21), S10Tnp-510 (n = 24), S10Tnp-1120 (n = 40), S10TnpC2+479(n = 21), ΔS10Tnp-510 (n = 21), ΔS10Tnp-1120 (n = 15), ΔS10TnpC2+479 (n = 11), S10Md(-510;-1120) (n = 15) or S10Md(-1120;C2+479) (n = 15) strains are shown. When the observed value was 0 CFU/fly points were plotted as 10^0^. Statistical significance was analyzed using Kruskal-Wallis non-parametric tests followed by Dunn’s multiple comparisons using parental as control respectively. n.s. stands for non-significant, p>0.05; *, p<0.05; **, p<0.01; ***, p<0.001;****, p<0.0001.

We next performed infection assays to compare parental and S10Tnp derivatives for host-invasion capacity at 48hs pi, when CFU within flies peak. Bacterial burden within flies reached similar levels when insects were infected with S10Tnp-35 or the parental strain (2210 and 5513 CFU/fly respectively). Infection with S10Tnp-510, S10Tnp-1120 and S10TnpC2+479, the derivatives displaying the lowest GR, showed a significantly lower bacterial burden. S10Tnp-510, S10Tnp-1120 and S10TnpC2+479 infections displayed median values of 0, 0 and 8 CFU/fly respectively (Fig 5). Fly invasion by the corresponding ΔS10Tnp derivatives, similarly to parental strain, showed bacterial burdens of 1280, 1310 and 4580 CFU/fly respectively. Finally, S10Md(-510;-1120) and S10Md(-1120;C2+479) were not affected in host-invasion capacity, showing a median of 3795 and 4600 CFU/fly respectively. These results show that in the otherwise isogenic strains S10-dosage plays an important role in host-colonization ability suggesting that it could constitute an important advantage for *V*. *cholerae* in its natural environment.

## Discussion

In all living organisms, DNA replication, expression of genetic information and the cell cycle are well coordinated. Bacterial systems have provided a great deal of information to understand this critical issue [50–53]. In this vein, few studies have linked gene location and bacterial physiology [4, 5, 13]. Experimental evidence is still scarce [36], mostly due to a lack of tools for precise relocation without widely altering genome structure. Here we employed a “positional genetics” approach in which the genes of interest are systematically relocated within the genome. This methodology provided insight into how gene location, and in particular the S10 locus, influence cell physiology.

Comparative genomics approaches showed that RP and RNAP genes in fast growing bacteria locate close to *oriC* suggesting a link between the genomic localization of these genes and cell physiology [16]. To our knowledge, the present work is the first in which this issue is directly assessed. We experimentally show that positioning of S10, a locus harboring half of the RP genes and the gene encoding for the alpha subunit of the RNAP, is essential for optimal growth and directly impact host-invasion capacity of *V*. *cholerae*. Nearby relocation did not show any detectable physiological effects. Upon repositioning S10 far away from *oriC1* at 4 different locations (Fig 1) we observed slower growth (Fig 2) and impaired host invasion capacity (Fig 5). S10 locus position is conserved in the *Vibrioneaceae* (S3 Table). Since the locus can be widely relocated along the genome (Fig 1) its current positioning is likely to be the result of strong selection in nature.

Previous *in silico* studies have proposed the gene-dosage hypothesis to explain the location bias for RP and RNAP [12, 16]. Two experimental approaches were used to show a bias on gene expression dependent on distance to *oriC*. First, several studies measured the phenotype given by an heterologous gene (i.e. *yfp*[8], *nahR*[54]) according to the position in which it was inserted within the genome [8, 12]. The second approach used high throughput techniques to show that genomic regions contiguous to *oriC* display higher expressiveness that decreases and reaches its minimum at the chromosome terminus [7, 30, 55]. Our positional genetics approach allowed for precise relocation of endogenous genes to study a key physiological function such as global growth control. This enabled us to assess the dosage and expression of genes (Fig 3B and 3C) as they are relocated and to correlate them to a phenotype (Fig 3D). In this work, we observed that S10 dosage, mRNA abundance and GR diminished the further away the locus was moved from *oriC1*. We report a strong co-variation between these three parameters (r>0.9). S10 seems to be already at an optimal genomic location since moving the locus towards *oriC1* had no effect on GR or S10 expression. In slow-growing conditions, when genes near *oriC1* do not benefit from a high increase in dosage [7, 44], we failed to detect significant GR variation among S10Tnp derivatives. This body of evidence strongly supports the gene-dosage hypothesis.

In this study, GR was measured after S10 transposition, which caused the simultaneous alteration of its position and dosage (Fig 3A). Therefore effects caused only by changes in dosage could not be distinguished from those generated by change in S10 location. Strains bearing two S10 copies far away from the *oriC1* allowed us to circumvent this problem by providing a higher copy number far from *oriC1*. In these mutants, GR in fast growing conditions and host-infection capacity were restored (Figs 4 and 5), strongly supporting the notion that S10 position is linked to cell physiology and that gene dosage is the main mechanism behind the observed positioning effects.

The genomic location of rDNA is biased towards the *oriC* in fast growing organisms [16]. Also, the number of rDNA operons per genome correlates to generation time [56]. A recent report by Gyorfy and colleagues [36] experimentally assessed the influence of copy number and location of rDNA on cell physiology in the model bacterium *E*. *coli*. The reduction in the number of rDNA operons led to a decrease in doubling time. Interestingly, the authors tested the addition of an extra rDNA gene copy at two different locations: close to *oriC* or far from it. In either case, they did not observe any GR increase. In the case of rDNA genes their high copy-number could mask any putative positional effect. Therefore, for these genes, dosage could be dominant over their genomic location. As we observed in our work, this was not the case for RP. Since they are in single copy, positional changes influence gene dosage in much greater proportion. A recent publication reported 300-fold expression changes of *gfp* according to its genomic site of insertion in slow growing conditions [25]. Such variation in expression cannot be accounted by gene dosage effects but rather the presence of overlapping chromosome structural organization features. Since we worked with some of the most highly expressed genes in the cell [30] and the observed S10 dosage differences correlated tightly to expression changes (Fig 3D), effects of local sequences on S10 expression are likely to be bypassed (Fig 4). As all these genes are essential [39] we think that small expression changes due to gene dosage reduction can influence growth rate. Considering this, S10’s case must not be unique and several genes involved in the flux of genetic information must face a similar scenario.

S10 proximity to *oriC1* might have additional benefits. By moving S10, its spatial address within the cell [1, 3, 27] is altered. It has been recently shown that after transcription, mRNA remains confined near its transcription site. This localization restricts ribosomal mobility and spatially organizes translation and mRNA decay [9, 27]. As mentioned before, drastic positional changes might physically separate S10 from functional partners such as rRNA operons and other RNAP and RP genes. Although our study does not rule out this possibility, at least in our experimental conditions, gene dosage appears to have a dominant effect.

Many ways of RP regulation [19–23], have been reported. In particular, S10 codes for L4, S8 and S4 which are regulatory RPs that control the expression of S10, spec and α loci respectively through a translational feedback mechanism [57]. Simultaneously, these proteins bind to rRNA molecules. Hence, rRNA availability controls RP abundance by countering translational feedback through direct competition for regulatory RP. It is known that, in exponential phase, rRNA transcription peaks. Hence, it can be reasonably assumed that S10 genes are at their maximum expression. This would permit gene dosage effects to be noticeable.

Regulation mechanisms controlling RP abundance have an impact on ribosome synthesis and regulate their abundance [23, 57, 58]. Similarly, RNAP regulation may influence global transcription [17]. Gene location can be considered a mechanism for RP and RNAP positive regulation during exponential growth having the benefit of up-regulating these functions specifically during exponential phase, the stage of the cell cycle where they are most needed. Our results lead us to propose that GR reduction might be the consequence of a reduction in the number of ribosomes and transcription foci, reducing total global expression capacity for the cell. Nevertheless, since RNAP α-subunit is considered to be in excess [17], we speculate that the observed effects are mainly a consequence of ribosomal function impairment. Additionally, it has been recently shown that RP expression coordinates chromosome replication with cell physiology by an *oriC* and DnaA-independent mechanism in *Bacillus subtillis* [59]. The authors observed a doubling time and ori/ter reduction when RP are repressed. This is in full concordance with our data (Figs 2 and 3), since on top of a reduced GR and S10 expression, we found a trend in ori1/ter1 ratio reduction when S10 is relocated along the chromosomes (Fig 3B, right panel). Furthermore, there is increasing evidence that RPs are involved in DNA replication control in several biological systems ranging from archaea and yeast to human cells and as such, being a putative universal mechanism coordinating cell cycle to cell physiological state [9, 60–62]. *V*. *cholerae* is a model organism for studying bacteria with multipartite genomes [63]. Fitness advantage of such genomic organization, occurring in 10% of known bacteria, remains poorly understood [41, 64]. Location of most of the essential genes in the main chromosome physically separates a stable set of genes from a plastic secondary chromosome that could serve as an evolutionary test bed [65]. Chromosomal location impacts gene expression as genes in secondary chromosomes are less expressed than those harbored by primary chromosomes [7, 30, 64, 65]. S10 is one of the most transcribed loci in *V*. *cholerae*, containing exclusively essential genes and is always found in bacterial main chromosomes [30, 37–39]. Genes linked to translation are under-represented in Chr2 [7]. Nevertheless, S10 transposition to Chr2 was viable. Within this replicon, dosage and expression also changed according to position (Figs 3 and S5). Since ori2/ter2 quotient remained constant in parental, S10TnpC2+37 and S10TnpC2+479 strains while S10/ter2 ratio showed a progressive reduction, we conclude that S10 expression differences are the result of dosage reduction. The latter is caused by Chr2 delayed replication with respect to Chr1 [29] combined to position-associated gene dosage reduction within this replicon. The high expression of the S10 locus renders detection of replication-associated gene-dosage effects possible, even along Chr2. In parallel, a constant ori2/ter2 ratio among these strains implies that replication regulation of Chr2 and coordination between both chromosomes is very tight, since over-firing *oriC2* or uncoupling Chr2 replication from Chr1 has the potential to compensate S10 dosage differences observed. In summary, here we show a novel method for testing the phenotypic impact of genome organization in bacteria. We showed a clear effect of S10 repositioning on *V*. *cholerae* GR and host invasion phenotypes. Application of this methodology to other bacteria and to other genes will provide insights into the rules of genome organization. Additionally, understanding the genomic factors affecting GR would permit to reprogram bacterial growth, help to predict the behavior of more complex biological systems, and develop better theoretical models [66, 67], thus promising a deep impact in genome design, bioengineering and biotechnology.

## Materials and Methods

### Bacterial strains and plasmids

Strains and plasmids used in this study are listed in S5 Table. Details of strain generation are in Supporting Information.

### RT-qPCR and qPCR assays

Strains cultured in LB at 37°C until OD_600nm_≈0.1 were chilled on ice and used to prepare gDNA and total RNA (see Supporting Information). RT of 2 μg of RNA was performed using SuperscriptIII (Invitrogen, MA, USA) and random hexamers in a 20 μL final volume. SYBR Green PCR Master Mix (Applied Biosystems MA, USA) was used according to manufacturer’s instructions. Primers are listed in S2 Table. Assays were performed on at least three different extracts in 10 μL volume per quadruplicate. In gene-dosage experiments, samples were normalized to a reference gDNA from a stationary-phase culture left at 4°C ON. In S10 expression measurements, cDNAs were serially diluted and the ΔΔC_T_ method was used for data analysis. The *gyrA*, *clpX* and *recA* were employed as reference genes while *rpsJ* was used to measure S10 expression. Only data in which C_T_ standard deviation was lower than 0.25 was used.

### *Drosophila melanogaster* infection assay

*Drosophila melanogaster w1118* flies were bred at 25°C. *V*. *cholerae* ON cultures were washed and diluted to 10^–4^ on 10% sucrose, streptomycin 100 μg/ml PBS solution. An innocuous blue dye was incorporated up to 2% v/v. Male flies were starved for 16 hours and placed in vials containing a filter paper embedded in 150μL of bacterial suspension. After 1 hour of feeding, flies showing a blue abdomen were kept. A first time point (0 h) was taken and the rest of the animals were transferred to new vials containing cotton plugs embedded in 10% sucrose, streptomycin 100 μg/ml PBS solution. Bacterial burden measurements were done by pestle-homogenizing 5 flies in 150 μL of PBS and plating in LB-agar with streptomycin. Colony counts were done after ON incubation at 30°C.

## Supporting Information

S1 Fig *Vibrio cholerae* chromosomes in scale showing bias towards *oriC1* of RP (green triangles), rRNA operons (red triangles) were created using GView [68]. S10 locus is magnified. Arrows show gene orientation and their sizes. Genes are colored according to their function.
(EPS)

S2 Fig Cell length distribution measured at exponential phase in fast-growth conditions by image analysis. CSLM images were analyzed using Image J. The length of at least 400 cells of each strain was measured. Parental and S10Tnp strains display similar cell length distribution.
(EPS)

S3 Fig Parental GR comparison in fast-growth conditions. Effect of attB’ insertion on GR was quantified by averaging the slope (μ) obtained in at least 5 independent experiments performed in quadruplicate for each parental strain. Results are expressed GR which is the mean μ with CI 95%. Statistical significance was analyzed using a one-way ANOVA two tailed test.
(EPS)

S4 Fig Parental and S10Tnp series GR comparison in slow growth conditions. Results of S10Tnp derivatives were normalized to parental strains and expressed as percentage of variation mean (μ %) with 95% CI with respect to parental strains. Statistical significance was analyzed using one-way ANOVA two tailed test. n.s. means non-significant difference.
(EPS)

S5 Fig S10 dosage and expression reduction is the consequence of gene dosage effects along Chr2. **(a)** Expected trend on S10/ter2 according to locus repositioning. Ellipses represent chromosomes. Colored dots depict *oriC1* and *oriC2* and termini of Chr1 (*ter1*) and Chr2 (*ter2*). Simultaneous replication rounds are shown. An orange arrow shows the S10 locus. **(b)** Gene dosage measurements obtained by qPCR in fast-growth conditions, showing the mean and error bars representing 95% CI. Statistical significance was assessed by one-way ANOVA two tailed test and Tukey’s test for multiple comparisons. The ori2/ter2 ratio quantifies replication of Chr2 while S10/ter2 quantifies S10 dosage.
(EPS)

S6 Fig S10 return to its original location restores growth rate. **(a)** S10 return to its original location, S10Tnp-1120 is shown as example. Left panel, an extra S10-copy at its original context (blue) is inserted by natural transformation in S10Tnp strain. Center and right panels, the transposed S10 (orange) is deleted by allelic replacement using *spec* ^R^ (pink) **(b)** Southern Blot using digested gDNA of the donor, parental, S10Tnp, S10Md and ΔS10Tnp strains of -1120 series. Probes were targeted to markers linked to donor *rpsJ* gene (green, DY782) or to the parental *rpsJ* gene (red, DY682). Genotype changes were evidenced by size change of S10 upon movement (parental vs S10Tnp), then donor allele insertion (S10Md) and parental allele deletion (ΔS10Tnp) **(c)** A representative growth curve and the mean % μ variation in the ensemble of experiments is plotted to observe complementation for each set of mutants as in Fig 2. Values were obtained from independent experiments (S7 Table). Statistical significance was assessed by one-way ANOVA two-tailed test. Tukey test was performed for multiple comparisons.
(EPS)

S7 Fig Time-lapse infection experiments. Flies are fed with the parental strain for one hour. Bacterial load is shown as CFU/fly at initial time (0), 24, 48 or 72hs after transferring flies to fresh tubes with no bacteria. Median is shown as a horizontal line. Statistical significance of differences was analyzed in both cases using Kruskal-Wallis non-parametric tests followed by Dunn’s multiple comparisons using initial load as control respectively. Results are shown as n.s., non significative difference, p>0.05; ****, p<0.0001.
(EPS)

S1 Table Ribosomal proteins within *s10-spec-alpha* locus. (DOCX)

S2 Table Ribosomal proteins in *Vibrio cholerae* genome not included in *s10-spec-alpha* locus. (DOCX)

S3 Table S10 genomic position is conserved among *Vibronaceae*. The table shows the 18 genomes analyzed, representing the three genera and the 12 species for which complete assemblies and *oriC* ^b^ coordinates are available.
(DOCX)

S4 Table Full list of plasmids, bacteria and *Drosophila melanogaster* strains used in this study. (DOCX)

S5 Table Absolute growth rates of strains generated in this study. (DOCX)

S6 Table Linear correlations of % μ variation, S10 dosage and expression with S10 position along the chromosomes. (DOCX)

S7 Table GR estimated by μ (min^-1^) obtained from automated culture experiments results for parental, S10Tnp and ΔS10Tnp comparison at the indicated locations within the genomes. These values were used in Fig 4C.
(DOCX)

S8 Table Oligonucleotides used in qPCR assays. (DOCX)

S1 Text Appendix: Supplementary Methods and Literature. (DOCX)

S1 Video Time-lapse microscopy of parental strain. Bacteria were distributed in an LB-agar layer kept at 37°C. Images of individual cells were recorded every 2 minutes.
(AVI)

S2 Video Time-lapse microscopy S10Tnp+166. Bacteria were distributed in an LB-agar layer kept at 37°C. Images of individual cells were recorded every 2 minutes.
(AVI)

S3 Video Time-lapse microscopy of S10Tnp-35 strain. Bacteria were distributed in an LB-agar layer kept at 37°C. Images of individual cells were recorded every 2 minutes.
(AVI)

S4 Video Time-lapse microscopy of S10Tnp-510 strain. Bacteria were distributed in an LB-agar layer kept at 37°C. Images of individual cells were recorded every 2 minutes.
(AVI)

S5 Video Time-lapse microscopy of S10Tnp-1120 strain. Bacteria were distributed in an LB-agar layer kept at 37°C. Images of individual cells were recorded every 2 minutes.
(AVI)

S6 Video Time-lapse microscopy of S10TnpC2+37 strain. Bacteria were distributed in an LB-agar layer kept at 37°C. Images of individual cells were recorded every 2 minutes.
(AVI)

S7 Video Time-lapse microscopy of S10TnpC2+479 strain. Bacteria were distributed in an LB-agar layer kept at 37°C. Images of individual cells were recorded every 2 minutes.
(AVI)
