# Supplementary material for: Genomic Location of the Major Ribosomal Protein Gene Locus Determines Vibrio cholerae Global Growth and Infectivity
Source: PLoS Genet. 2015 Apr 13;11(4):e1005156. doi: 10.1371/journal.pgen.1005156 (PMC4395360; doi:10.1371/journal.pgen.1005156)
Supplement: S1 Table — (DOCX) [file pgen.1005156.s008.docx]

**S1 Table**. Ribosomal proteins within *s10-spec-alpha* locus

| Gene name | Locus tag | Product |
| --- | --- | --- |
| *rplQ* | VC2570 | 50S ribosomal protein L17 |
| *rpsD* | VC2572 | 30S ribosomal protein S4 |
| *rpsK* | VC2573 | 30S ribosomal protein S11 |
| *rpsM* | VC2574 | 30S ribosomal protein S13 |
| *rpmJ* | VC2575 | 50S ribosomal protein L36 |
| *rplO* | VC2577 | 50S ribosomal protein L15 |
| *rpmD* | VC2578 | 50S ribosomal protein L30 |
| *rpsE* | VC2579 | 30S ribosomal protein S5 |
| *rplR* | VC2580 | 50S ribosomal protein L18 |
| *rplF* | VC2581 | 50S ribosomal protein L6 |
| *rpsH* | VC2582 | 30S ribosomal protein S8 |
| *rpsN* | VC2583 | 30S ribosomal protein S14 |
| *rplE* | VC2584 | 50S ribosomal protein L5 |
| *rplX* | VC2585 | 50S ribosomal protein L24 |
| *rplN* | VC2586 | 50S ribosomal protein L14 |
| *rpsQ* | VC2587 | 30S ribosomal protein S17 |
| *rpmC* | VC2588 | 50S ribosomal protein L29 |
| *rplP* | VC2589 | 50S ribosomal protein L16 |
| *rpsC* | VC2590 | 30S ribosomal protein S3 |
| *rplV* | VC2591 | 50S ribosomal protein L22 |
| *rpsS* | VC2592 | 30S ribosomal protein S19 |
| *rplB* | VC2593 | 50S ribosomal protein L2 |
| *rplW* | VC2594 | 50S ribosomal protein L23 |
| *rplD* | VC2595 | 50S ribosomal protein L4 |
| *rplC* | VC2596 | 50S ribosomal protein L3 |
| *rpsJ* | VC2597 | 30S ribosomal protein S10 |
