# Supplementary material for: Genomic Location of the Major Ribosomal Protein Gene Locus Determines Vibrio cholerae Global Growth and Infectivity
Source: PLoS Genet. 2015 Apr 13;11(4):e1005156. doi: 10.1371/journal.pgen.1005156 (PMC4395360; doi:10.1371/journal.pgen.1005156)
Supplement: S2 Table — (DOCX) [file pgen.1005156.s009.docx]

| Gene name | Locus tag | Product | Location | Orientation^1^ |
| --- | --- | --- | --- | --- |
| *rpmH* | VC0007 | 50S ribosomal protein L34 | Chromosome 1 | LS |
| *rpmB* | VC0218 | 50S ribosomal protein L28 | Chromosome 1 | RS |
| *rpmG* | VC0219 | 50S ribosomal protein L33 | Chromosome 1 | RS |
| *rplK* | VC0324 | 50S ribosomal protein L11 | Chromosome 1 | RS |
| *rplA* | VC0325 | 50S ribosomal protein L1 | Chromosome 1 | RS |
| *rplJ* | VC0326 | 50S ribosomal protein L10 | Chromosome 1 | RS |
| *rplL* | VC0327 | 50S ribosomal protein L7/L12 | Chromosome 1 | RS |
| *rpsL* | VC0359 | 30S ribosomal protein S12 | Chromosome 1 | RS |
| *rpsG* | VC0360 | 30S ribosomal protein S7 | Chromosome 1 | RS |
| *rpsF* | VC0366 | 30S ribosomal protein S6 | Chromosome 1 | RS |
| *rpsR* | VC0368 | 50S ribosomal protein S18 | Chromosome 1 | RS |
| *rplI* | VC0369 | 50S ribosomal protein L9 | Chromosome 1 | RS |
| *rplU* | VC0435 | 50S ribosomal protein L21 | Chromosome 1 | RS |
| *rpmA* | VC0436 | 50S ribosomal protein L27 | Chromosome 1 | RS |
| *rpsU* | VC0520 | 30S ribosomal protein S21 | Chromosome 1 | LS |
| *rpsP* | VC0561 | 30S ribosomal protein S16 | Chromosome 1 | RS |
| *rplS* | VC0564 | 50S ribosomal protein L19 | Chromosome 1 | RS |
| *rplM* | VC0570 | 50S ribosomal protein L13 | Chromosome 1 | RS |
| *rpsI* | VC0571 | 30S ribosomal protein S9 | Chromosome 1 | RS |
| *rpsO* | VC0646 | 30S ribosomal protein S15 | Chromosome 1 | RS |
| *rpsT* | VC0679 | 30S ribosomal protein S20 | Chromosome 1 | LS |
| *rpmE2* | VC0878 | 50S ribosomal protein L31 | Chromosome 1 | RS |
| *rpmJ** | VC0879 | 50S ribosomal protein L36 | Chromosome 1 | RS |
| *rplY* | VC1640 | 50S ribosomal protein L25 | Chromosome 1 | LS |
| *rpsA* | VC1915 | 30S ribosomal protein S1 | Chromosome 1 | RS |
| *rpmF* | VC2025 | 50S ribosomal protein L32 | Chromosome 1 | RS |
| *rpsB* | VC2260 | 30S ribosomal protein S2 | Chromosome 1 | RS |
| *rpmE* | VC2679 | 50S ribosomal protein L31 | Chromosome 1 | LS |
| *rpmI* | VCA0289 | 50S ribosomal protein L35 | Chromosome 2 | RS |
| *rplT* | VCA0290 | 50S ribosomal protein L20 | Chromosome 2 | RS |

^1^RS, Replication Sense, the gene is in located on the replicative leading strand avoiding negative effects caused by collisions between replication and transcription machineries. LS, Lagging Strand, the gene is transcribed in the opposite direction to replication, and then gene transcription may be a source of replication-transcription conflicts. *similar to the one found within *S10-spec-α* locus (40% identical, 60% similar).
