# Supplementary material for: Genomic Location of the Major Ribosomal Protein Gene Locus Determines Vibrio cholerae Global Growth and Infectivity
Source: PLoS Genet. 2015 Apr 13;11(4):e1005156. doi: 10.1371/journal.pgen.1005156 (PMC4395360; doi:10.1371/journal.pgen.1005156)
Supplement: S3 Table — The table shows the 18 genomes analyzed, representing the three genera and the 12 species for which complete assemblies and oriC b coordinates are available. (DOCX) [file pgen.1005156.s010.docx]

| ***Vibrionaceae* species^a^** | **Ref. sequence** | ***rpsJ* tag** | **S10 distance to *oriC1*(bp)*^b^*** | **S10 position (%replichore)^c^** |
| --- | --- | --- | --- | --- |
| *Vibrio anguillarum 772* | NC_015633 | VAA_02669 | 403117 | 26,3 |
| *Vibrio cholerae* N16961 | NC_002505 | VC2597 | 196577 | 13,2 |
| *Vibrio cholerae* M66-2 | NC_012578 | VCM66_2518 | 190828 | 13,2 |
| *Vibrio cholerae* MJ-1236 | NC_012668 | VCD_01766 | 312140 | 19,8 |
| *Vibrio cholerae* O395 | NC_009457 | VC095_A2175 | 350606 | 23,2 |
| *Vibrio furnissii* NCTC 11218 | [NC_016602](http://www.ncbi.nlm.nih.gov/nuccore/NC_016602.1) | VFU_A00666 | 350351 | 21,2 |
| *Vibrio harveyi* ATCC BAA-1116 **^d^** | [NC_009783](http://www.ncbi.nlm.nih.gov/nuccore/NC_009783.1) | VIBHAR_00728 | 305538 | 16,2 |
| *Vibrio parahaemolyticus* RIMD 2210633 | NC_004603 | VP0256 | 267342 | 16,2 |
| *Vibrio sp.* Ex25 | NC_013456 | VEA_001743 | 298546 | 18,3 |
| *Vibrio splendidus* LGP32 **^e^** | [NC_011753](http://www.ncbi.nlm.nih.gov/nuccore/NC_011753.2) | VS_2833 | 326852 | 19,8 |
| *Vibrio vulnificus* MO6-24/O | NC_014965 | VVMO6_02756 | 240620 | 15,1 |
| *Vibrio vulnificus* YJ016 | NC_005139 | VV0374 | 375184 | 22,3 |
| *Vibrio vulnificus* CMCP6 | NC_004459 | VV1_0763 | 247081 | 15,1 |
| *Vibrio sp.* Strain EJY3 | NC_016613 | VEJY3_01275 | 288360 | 16,6 |
| *Aliivibrio fischeri* ES114 | NC_006840 | VF_0234 | 2652840 | 17,4 |
| *Aliivibrio fischeri* MJ11 | NC_011184 | VFMJ11_0224 | 242508 | 16,7 |
| *Aliivibrio salmonicida LFI1238* | NC_011312 | VSAL_I0319 | 363246 | 21,8 |
| *Photobacterium profundum SS9* | NC_006370 | PBPRA0319 | 332539 | 16,3 |

**^a^**representative *Vibrionaceae* species were selected among those whose genome is completely assembled. **^b^**The *oriC1* was coordinates were obtained from *DoriC* Database[[69](#_ENREF_2)]. Distance was taken from *oriC1* to *rpsJ*. **^c^** Distance between S10 and *oriC1* was divided by the replichore length and multiplied by 100. **^d^** Now known as *Vibrio campbelli*. **^e^** Now known as *Vibrio tasmaniensis*.
