# Supplementary material for: Genomic Location of the Major Ribosomal Protein Gene Locus Determines Vibrio cholerae Global Growth and Infectivity
Source: PLoS Genet. 2015 Apr 13;11(4):e1005156. doi: 10.1371/journal.pgen.1005156 (PMC4395360; doi:10.1371/journal.pgen.1005156)
Supplement: S4 Table — (DOCX) [file pgen.1005156.s011.docx]

| **Name** | **Relevant genotype or features** | **Reference** |
| --- | --- | --- |
| **Plasmids** | | |
| pMP96 | pSC101*rep*^TS^ *oriT*_RP4_ [*int_λ_-xis_λ_, int_HK_-xis_HK_*] | **[**[**3**](#_ENREF_3)**]** |
| pCP20 | pSC101*rep*^TS^ [*flp*] | [[4](#_ENREF_4)] |
| pASB6 | pCR-BluntII-Topo:: lox2272-ere(A)-lox2272 | This study |
| pASB9 | pCR-BluntII-Topo:: lox66-*dfrB1*-lox71 | This study |
| pASB11 | pCR-BluntII-Topo:: lox66-Zeo-lox71 | This study |
| pMP99 | oriRK6 [*attR_HK_- bla*^R^’-*FRT-aph-FRT*] *cat* | This study |
| pMP101 | oriRK6 [*bla*^R^’-*attR_HK_-FRT-cat-FRT*] *aph* | This study |
| ***Escherichia coli*** | | |
| DH5α | F^-^ endA1 glnV44 thi-1 recA1 relA1 gyrA96 deoR nupG Φ80d*lacZ*ΔM15 Δ(*lacZYA-argF*)U169, hsdR17(r_K_^-^ m_K_^+^), λ^–^ |  |
| ***Vibrio cholerae*** | | |
| N16961 | *Vibrio cholerae* serotype O1 biotype El Tor strain N16961 | [[5](#_ENREF_5)] |
| N16961*ΔlacZ* | N16961 Δ*lacZ* | [[3](#_ENREF_3)] |
| N16961*ChapR* | N16961 | [[6](#_ENREF_6)] |
| N16961*ChapRΔlacZ* | N16961::mTn*7hapR^+^* Δ*lacZ* using pMEV69 | [[3](#_ENREF_3)] |
| PGB-A192 | N16961 Δ*lacZ*, [3'*blaR-attL_HK022_*-*FRT*-*aph*-*FRT*] (at the intergenic space VC2569-VC2570), [*FRT*-*cat*-*FRT*-*attR_HK022_*-5'*blaR*] (at the intergenic space VC2599-VC2600) flanking the region VC2569 and VC2599 encompassing *S10-spc-α locus*. | This study |
| Parental +166 | ASB-A192::*attB*’-lox66-*dfrB1*-lox71 inserted in the intergenic region between VC2739-VC2740 | This study |
| Parental -35 | ASB-A192::*attB*’-*lox66-dfrB1-lox71* inserted in the intergenic region between VC2536-VC2537. | This study |
| Parental -510 | PGB-A192::*attB*’*-lox66-dfrB1-lox71* inserted in the intergenic region between VC2075-VC2076. | This study |
| Parental -1120 | PGB-A192::*attB*’-*lox66-dfrB1-lox71* inserted in the intergenic region VC1508-VC1509. | This study |
| Parental -1120  Δ(*aph,cat*) | PGB-A192::*attB*’*-lox66-dfrB1-lox71* Δ(*aph,cat*) inserted in the intergenic region VC1508-VC1509. Kanamycin and chloramphenicol resistance cassettes were deleted using a flipase expressing plasmid. | This study |
| Parental C2+37 | PGB-A192::*attB*’*-lox66-dfrB1-lox71* inserted in the intergenic region between VCA0030-VCA0031. | This study |
| Parental C2+479 | PGB-A192::*attB*’*-lox66-dfrB1-lox71* inserted in the intergenic region between VCA0543-VCA0544. | This study |
| S10Tnp+166 | S10 relocated closer to *oriC1*. Derived from Parental+166. | This study |
| S10Tnp-35 | S10 relocated next to its original location. Derived from Parental-35. | This study |
| S10Tnp-510 | S10 relocated at the middle of the left replichore of chromosome 1. Derived from Parental-510. | This study |
| S10Tnp-1120 | S10 relocated near the *dif* region of chromosome 1. Derived from Parental -1120. | This study |
| S10TnpC2+37 | S10 relocated near the *oriC2*. Derived from Parental C2+479. | This study |
| S10TnpC2+479 | S10 relocated near the *dif* sequence of chromosome 2. Derived from Parental C2+479. | This study |
| PGB-B393 | N16961 Δ*lacZ*, [lox2272-*ere(A)*-lox2272] (at the intergenic space VC2569-VC2570), [*lox66*-*zeo^R^*-*lox71*] (at the intergenic space VC2599-VC2600) flanking the region VC2569 and VC2599 encompassing *S10-spc-α* locus. | This study |
| S10Md-35 | Merodiploid bearing two *S10-spc-α* copies. Additional copy inserted in the intergenic region between VC2536-2537. | This study |
| S10Md-510 | Merodiploid bearing two *S10-spc-α* copies. Additional copy inserted in the intergenic region between VC2075-VC2076. | This study |
| S10Md-1120 | Merodiploid bearing two *S10-spc-α* copies. Additional copy inserted in the intergenic region between VC1508-VC1509. | This study |
| S10MdC2+479 | Merodiploid bearing two *S10-spc-α* copies. Additional copy inserted in the intergenic region between VCA0543-VCA0544. | This study |
| ΔS10Tnp-35 | N16961 Δ*lacZ*, S10 in single copy. *ere(A)*- *S10-spc-α* locus -*zeo^R^* encompassing. Spec^R^ gene in the intergenic region between VC2536-2537. | This study |
| ΔS10Tnp-510 | N16961 Δ*lacZ*, S10 in single copy. *ere(A)*- *S10-spc-α* locus -*zeo^R^* encompassing. Spec^R^ gene in the intergenic region between VC2075-VC2076. | This study |
| ΔS10Tnp-1120 | N16961 Δ*lacZ*, S10 in single copy. *ere(A)*- *S10-spc-α* locus -*zeo^R^* encompassing. Spec^R^ gene in the intergenic region between VC1508-VC1509. | This study |
| ΔS10TnpC2+37 | N16961 Δ*lacZ*, S10 in single copy. *ere(A)*- *S10-spc-α* locus -*zeo^R^* encompassing. Spec^R^ gene in the intergenic region between VCA0030-VCA0031. |  |
| ΔS10TnpC2+479 | N16961 Δ*lacZ*, S10 in single copy. *ere(A)*- *S10-spc-α* locus -*zeo^R^* encompassing. Spec^R^ gene in the intergenic region between VCA0543-VCA0544. | This study |
| S10Md(-510,-1120) | Merodiploid bearing *S10-spc-α* copies at the intergenic sequences of VC2075-VC2076 and VC1508-VC1509. | This study |
| S10Md(-1120,C2+479) | Merodiploid bearing *S10-spc-α* copies at the intergenic sequences of VC1508-VC1509 and VCA0543-VCA0544. | This study |
| ***Drosophila melanogaster*** | | |
| w^1118^ | White eye phenotype | FlybaseID:  FBal0018186 |
