# Supplementary material for: Genomic Location of the Major Ribosomal Protein Gene Locus Determines Vibrio cholerae Global Growth and Infectivity
Source: PLoS Genet. 2015 Apr 13;11(4):e1005156. doi: 10.1371/journal.pgen.1005156 (PMC4395360; doi:10.1371/journal.pgen.1005156)
Supplement: S5 Table — (DOCX) [file pgen.1005156.s012.docx]

| Strain | Fast Growth | Slow Growth |
| --- | --- | --- |
|  | μ (min^-1^) ^a^,^b^ | μ (min^-1^) ^a,b^ |
| Parental | 0.01825 ± 0.00018-Ref. | 0.00472 ± 0.00063-Ref. |
| S10Tnp+166 | 0,01838 ± 0.00021 n.s. | 0.00432 ± 0.00033 n.s. |
| S10Tnp-35 | 0.01822 ± 0.00023 n.s. | 0.00493 ± 0.00052 n.s. |
| S10Tnp-510 | 0.01718 ± 0.00022 *** | 0.00472 ± 0.00056 n.s. |
| S10Tnp-1120 | 0.01536 ± 0.00044 **** | 0.00437 ± 0.00056 n.s. |
| S10TnpC2+37 | 0.01729 ± 0.00022 *** | 0.00461 ± 0.00059 n.s. |
| S10TnpC2+479 | 0.01511 ± 0.00028 **** | 0.0044 ± 0.00067 n.s. |

^a^Directly obtained from slopes of growth curves during exponential phase. ^b^ Statistical significance is assessed using one-way ANOVA two-tailed test and Dunnetts’s test for multiple comparisons using parental as control. Ref, reference strain; ns, non-significant, p>0.05; ***, p<0.001; ****, p<0.0001.
