# Supplementary material for: Genomic Location of the Major Ribosomal Protein Gene Locus Determines Vibrio cholerae Global Growth and Infectivity
Source: PLoS Genet. 2015 Apr 13;11(4):e1005156. doi: 10.1371/journal.pgen.1005156 (PMC4395360; doi:10.1371/journal.pgen.1005156)
Supplement: S6 Table — (DOCX) [file pgen.1005156.s013.docx]

|  | % variation of μ | % of S10 dosage | % of S10 expression |
| --- | --- | --- | --- |
| **Linear equation** | Y=-0,1815*X + 2,131 | Y=-0,7402*X + 111,8 | Y=-0,4827*X + 104 |
| **R^2^** | 0.8948 | 0.9550 | 0.9822 |
