# Supplementary material for: Genomic Location of the Major Ribosomal Protein Gene Locus Determines Vibrio cholerae Global Growth and Infectivity
Source: PLoS Genet. 2015 Apr 13;11(4):e1005156. doi: 10.1371/journal.pgen.1005156 (PMC4395360; doi:10.1371/journal.pgen.1005156)
Supplement: S7 Table — These values were used in Fig 4C. (DOCX) [file pgen.1005156.s014.docx]

| **Chromosome 1** | -35 | -510 | -1120 |
| --- | --- | --- | --- |
| Parental | 0.01846 ± 0.00012 | 0.01813 ±  0.00027 | 0.018 ± 0.0002 |
| ΔS10Tnp* | 0.018 ±  0.000749166 | 0.0174 ±  0.0002 | 0.018 ± 0.0004 |
| S10Tnp | 0.01825 ±  0.000387657 | 0.01672 ± 0.00032 | 0.0156 ± 0.0006 |
| N | 5 | 7 | 5 |

| **Chromosome 2** | C2+37 | C2+497 |
| --- | --- | --- |
| Parental | 0.01854 ± 0.0004 | 0.018 ± 0.00018 |
| ΔS10Tnp* | 0,01795 ± 0,0003 | 0.01778 ± 0.00022 |
| S10Tnp | 0.017±  0.001 | 0.01554 ± 0.00029 |
| N | 6 | 5 |

*at least three independent clones were tested and averaged
