# Supplementary material for: Genomic Location of the Major Ribosomal Protein Gene Locus Determines Vibrio cholerae Global Growth and Infectivity
Source: PLoS Genet. 2015 Apr 13;11(4):e1005156. doi: 10.1371/journal.pgen.1005156 (PMC4395360; doi:10.1371/journal.pgen.1005156)
Supplement: S8 Table — (DOCX) [file pgen.1005156.s015.docx]

| Primer name | Sequence | Target | Use |
| --- | --- | --- | --- |
| S10 qPCR_F | AAATCGTTGAAACAGCTAAGCG | VC2597, *rpsJ* | S10 dosage and expression |
| S10 qPCR_R | GCATCTTTGTTAACGTGTGGAG | VC2597, *rpsJ* | S10 dosage and expression |
| VCori1Fw | GATTCATTACGCCGCTTCTCCC | VC2775, *gidA* | S10 dosage |
| VCori1Rev | TGAAGCTCAATGCGGCTAAACC | VC2775, *gidA* | S10 dosage |
| Ter1qPCR_Fw | TTCTTGAAGCCTCGCTGAGTAG | VC1410 | S10 dosage |
| Ter1qPCR_Rev | CGTAAGCGCCACTTTAATGCAG | VC1410 | S10 dosage |
| VCori2Fw | AGGCCTTCTCGGTATCCGTCTC | VCA0003 | S10 dosage |
| VCori2Rev | CTGCCGTCGCTGAATTACAACC | VCA0003 | S10 dosage |
| VC ter2 Fw | AGCGCCGCCTATTCAGGTATCC | VCA0563 | S10 dosage |
| VC ter2 Rev | GACAGAAGCTACCCACGCAAAC | VCA0563 | S10 dosage |
| gyrA Fwd | GGGTGTGGTTTCGATCAAAGTC | VC1258, *gyrA* | S10 expression |
| gyrA Rev | ACGTACCAGTGTACCGGCATTG | VC1258, *gyrA* | S10 expression |
| qRT_clpX_fwd | GTTCGCAAACTGATCGCAGG | VC1921, *clpX* | S10 expression |
| qRT_clpX_rev | CGGCTAGCACTTTTTTGGCG | VC1921, *clpX* | S10 expression |
| recA qFwd | TGTCTCTGGATATCGCGTTGGG | VC0543, recA | S10 expression |
| recA qRev | GTTGTGCAGCAGCAATCAGTTC | VC0543, recA | S10 expression |
