# Supplementary material for: Genomic Location of the Major Ribosomal Protein Gene Locus Determines Vibrio cholerae Global Growth and Infectivity
Source: PLoS Genet. 2015 Apr 13;11(4):e1005156. doi: 10.1371/journal.pgen.1005156 (PMC4395360; doi:10.1371/journal.pgen.1005156)
Supplement: S1 Text — (DOCX) [file pgen.1005156.s016.docx]

**General procedures.** Genomic DNA was extracted using the GeneJET Whole Blood Genomic DNA Purification Kit while plasmid DNA was extracted using the GeneJET Plasmid Miniprep Kit (Thermo Scientific, Lafayette, CO, USA). Total RNA was isolated using the High Pure RNA Isolation Kit (Roche, IN, USA). RNA quality was verified by gel electrophoresis and by spectrophotometry (A_260_/A_280_ and A_260_/A_230_ ratios) on a NanoDrop ND1100(Thermo Scientific, Lafayette, CO, USA). PCR assays were performed using Phusion High-Fidelity PCR Master Mix (Thermo Scientific, Lafayette, CO, USA). Radioactivity was visualized using Amersham Hyperfilm MP (GE Healthcare Europe GmbH, Velizy-Villacoublay, France). Fluorescent probes were detected with detection using the Odyssey infrared imaging system (LI-COR Biosciences, NE, USA).

**Culture conditions.** Bacterial cultures were done in Lennox Luria broth (LB) unless stated otherwise with appropriate antibiotics. Chloramphenicol (Cm, 3 μg/mL), kanamycin (Kan, 25 μg/mL), spectinomycin (Spec, 100 μg/mL ), carbenicillin (Carb, 50 μg/mL), erythromycin ( ere,12 μg/mL) and zeocin (Zeo,25 μg/mL) were added when required. When trimethoprim (Tmp 100 μg/mL) selection was applied, Mueller-Hinton medium was used.

**Modification of the attB’ sequences embedded within *bla* reporter.**

Full attB_HK_ site (wild type sequence, reverse and complement)

ATTCACGGTCGGTGCACTTT AGGTGAA AAAGGTTGAGTCGCAAAGCGGAAT 51bp

Mutated attB_HK_ embedded within *bla*

CACTTT AGGTGAA AAAGGTTGTG 23bp

Used sequence for attB’ sites

GTGCACTTT AGGTGAA AAAGGTTGAGTC 28bp

**S10Tnp generation.** The S10 locus in *V. cholerae* encompasses CDs VC2570 to VC2597. The attL_HK022_ was introduced between VC2569 and VC2570 and attR _HK022_ site between VC2599 and VC2600 using natural transformation [[73](#_ENREF_7),[74](#_ENREF_8)]. These sites linked to antibiotic resistant markers were amplified from plasmids pMP101 and pMP99 (S4 Table). After natural transformation with these DNA fragments, PGB-A192 strain was generated. A primer encoding a 28bp attB’ site was designed to amplify a third antibiotic resistant marker from pASB9 and then joined to upstream and downstream homology regions by PCR assembly for each target region (S4 Table). In all cases, attB’ sites were placed at intergenic zones of inversely oriented ORFs to avoid gene or operon interruption. Upon PGB-A192 transformation with these DNA fragments, parental strains bearing attL, attR and attB’ were generated (S4 Table, Figure 1b). Each parental strain was transformed with plasmid pMP96 and incubated in LB at 30°C until reaching an OD_600nm_ of 0.3-0.5. Plating on carbenicillin-supplemented LB-agar allowed recovery of Carb^R^ Spec^S^ colonies. Candidates were confirmed as S10Tnp mutants if they met the following criteria i) Carb^R^ phenotype ii) PCR: *bla* amplification, iii) PCR: attB’ absence and iv) Southern Blot showing the expected size change in the restriction fragment. Several clones were conserved at -80°C and were minimally sub-cultured to avoid emergence of suppressor clones.

**S10Md and ΔS10Tnp generation.** The *V. cholerae* S10 locus was flanked with two markers. The zeocin resistance gene was introduced between VC2599 and VC2600 and *ere(A)* [[75](#_ENREF_9)], was inserted between VC2569 and VC2570 as previously described. Markers were amplified from pASB11 and pASB6 plasmids respectively (S4 Table). Genomic DNA from this strain was used to transform each S10Tnp mutant. Zeo^R^ and Ere^R^ clones were selected. Merodiploidy was confirmed by Southern Blot. Spec^R^ gene was PCR-amplified and joined by PCR assembly to 1.5 Kbp homology regions upstream and downstream of each S10Tnp. DNA fragment was used to transform S10Md strains. Spec^R^, Cm^S^ and Km^S^ candidates were confirmed by amplification of *spec^R^* linkage to chromosome and Southern Blot.

**S10Md(-510;-1120) and S10Md(-1120;C2+479) generation.** Parental-1120 strain was transformed with pCP20 plasmid and selected on LB agar plates supplemented with carbenicillin. Transformed clones were cultured ON at 30°C for *aph* and *cat* removal by recombination reaction catalyzed by Flp. Then, individual clones were selected from LB-agar plates and restreaked on plates supplemented with kanamycin, with chloramphenicol and LB. Clones showing a Kan^S^ Cm^S^ phenotype were then grown ON on liquid LB at 37°C to promote pCP20 plasmid loss. Culture was restreaked on LB-agar plates to obtain isolated clones. Those that were susceptible to carbenicillin were selected for S10 transposition as described before (see S10Tnp generation). As expected, this strain showed the same phenotypes that the formerly obtained S10Tnp-1120 (Figures 4 and 5). Selected strains were induced for natural compentence[[7](#_ENREF_7)3,[74](#_ENREF_8)] and transformed either with gDNA from S10Tnp-510 or with gDNA from S10TnpC2+479. Cm^R^ and Kan^R^ clones were selected on LB-agar plates supplemented with the adequate antibiotics. Genotype was further confirmed by Southern Blot.

**Southern Blot.** DNA was extracted from 0.4 mL of an ON bacterial culture. DNA was dried on a speedvac device and then was digested with the appropriate restriction enzymes (*Eco*RV or *Hind*III). Samples were resolved by gel electrophoresis in 1X TAE buffer. After drying the gel for 1h at 60°C, DNA was denatured by rinsing in Denaturation Solution, NaOH 0.5 M, NaCl 0.15 M for 20 minutes at room temperature. The gel was equilibrated using Neutralization Solution, Tris 0.5 M pH=8 NaCl 0.15 M for 20 min. Then pre-hybridization was performed for 30 minutes at 50°C with 10 mL of RapidHyb Buffer (GE). Next, hybridization was performed ON at 50°C with a 40 nucleotide long DNA probe labeled in 5’ using T4 polynucleotide kinase following the manufacturer’s instructions and purified using a G-25 column (GE). Additionally, fluorescent probes were designed by coupling oligonucleotides to DY682 or DY782 dyes. Finally, washes were performed once in 6X SSC, 0.5% SDS at 50°C, and twice for 20 minutes at 55°C. A final wash was performed for 15 minutes at 60°C. Then gel was then placed between two Whatman 3MM papers and Saran wrap and developed on Kodak Film in the case of radioactive probes. For fluorescent probes Odyssey infrared imaging system was used.

Probes used:

S10hybr5’: targeting *rpsJ* gene.

5’-TCACGACTCGGAATTGGATTGTCATAATTGTCGGCTTCGC-3’

ZeoDY782_X2: targeting zeo^R^ gene that is linked to *rpsJ*.

  5'DY782-CAAAATCATCTTCCACAAAATCGCGGCTAAAACCCAGACG-3’ DY782

Cat-DY682_X2: targeting *cat* gene that is linked to *rpsJ*.

  5'DY682-GTATTCATTAAGCATCTGCCGACATGGAAGCCATCACAAA-3’ DY682

**CLSM Microscopy.** The indicated strains were grown to early exponential phase in LB, up to OD_600_≈0.2. Then cells were washed by centrifugation and resuspended in 50 μL of saline buffer. Then 5 μL of a 50 μg/mL FM5-95 stock solution was added and cells were placed on ice for 10 minutes. Cells were washed again and fixed by resuspending cells in 2% formaldehyde PBS solution and incubating. After 15 minutes of incubation at room temperature bacteria were washed, resuspended in 25 μL of PBS and then poured on 2% agarose saline buffer and covered. Images were taken by confocal laser scanning microscopy using a Leica-TCS-SP5 confocal microscope (Leica, Wetzlar, Germany). Image analysis was done using Image J.

**Time-lapse microscopy.** The indicated strains were grown in LB to OD_600_≈0.5 and diluted 1/300. Then, 3 μL were distributed in a LB-agar layer poured on a Gene Frame (1.5x1.6, Thermo Scientific, CO, USA) and covered with a glass cover slip. By removing part of the agar layer, two strains could be processed at a time while providing air. Next, samples were observed on a Zeiss AxioVison inverted microscope by phase contrast (Zeiss, Oberkochen, Germany). Microscope was equipped with a heat chamber which permitted to keep sample temperature at 37°C while imaging. Finally, 10 fields containing isolated cells were selected and each one was photographed every 2 minutes for at least 3 ho urs. Image series were analyzed using AxioVision (Zeiss, Germany) and Image J software.

**Growth curves**. ON cultures of the indicated microorganism were washed twice in PBS. For fast-growing conditions, the cell suspension was diluted 1/1000 in LB and incubated at 37°C during the experiment. For slow-growing conditions, the bacterial suspension was diluted 1/100 in M9 minimal medium supplemented with 1% fructose, 1mM MgSO_4_, 0.2 mM CaCl_2_ and incubated at 30°C during the experiment. Bacterial preparations were distributed by triplicate or quadruplicate in p96 microplates. Growth-curve experiments were performed using a TECAN Inﬁnite 200 microplate reader (TECAN, Männedorf, Germany), with absorbance (620nm) taken at 5-minutes intervals for a period of 12 h. The obtained OD values were plotted in time and using Microsoft Excel in logarithmic scale. The linear part of the curve was used to estimate μ from the slope of R^2^>0.995.

**Supplementary References**

68. Petkau A, Stuart-Edwards M, Stothard P, Van Domselaar G (2010) Interactive microbial genome visualization with GView. Bioinformatics 26: 3125-3126.

69. Gao F, Luo H, Zhang CT (2013) DoriC 5.0: an updated database of oriC regions in both bacterial and archaeal genomes. Nucleic Acids Res 41: D90-93.

70. Val ME, Skovgaard O, Ducos-Galand M, Bland MJ, Mazel D (2012) Genome engineering in *Vibrio cholerae*: a feasible approach to address biological issues. PLoS Genet 8: e1002472.

71. Stokke C, Waldminghaus T, Skarstad K (2011) Replication patterns and organization of replication forks in Vibrio cholerae. Microbiology 157: 695-708.

72. Heidelberg JF, Eisen JA, Nelson WC, Clayton RA, Gwinn ML, et al. (2000) DNA sequence of both chromosomes of the cholera pathogen Vibrio cholerae. Nature 406: 477-483.

73. Meibom KL, Blokesch M, Dolganov NA, Wu CY, Schoolnik GK (2005) Chitin induces natural competence in Vibrio cholerae. Science 310: 1824-1827.

74. De Souza Silva O, Blokesch M (2010) Genetic manipulation of Vibrio cholerae by combining natural transformation with FLP recombination. Plasmid 64: 186-195.

75. Marvig RL, Blokesch M (2010) Natural transformation of Vibrio cholerae as a tool--optimizing the procedure. BMC Microbiol 10: 155.

76. Biskri L, Mazel D (2003) Erythromycin esterase gene ere(A) is located in a functional gene cassette in an unusual class 2 integron. Antimicrob Agents Chemother 47: 3326-3331.
